# Smooth Surface Visual Imaging Method for Eliminating High Reflection Disturbance

**DOI:** 10.3390/s19224953

**Published:** 2019-11-14

**Authors:** Wei Shao, Kaibin Liu, Yunqiu Shao, Awei Zhou

**Affiliations:** 1 Faculty of Mechanical and Precision Instrument Engineering, Xi’an University of Technology, Xi’an 710048, Shanxi, China; kaibinliu@163.com (K.L.); s1017078041@outlook.com (Y.S.); 2 School of Mechanical and Electrical Engineering, Xi’an Polytechnic University, Xi’an 710048, Shanxi, China; lxmwsawz@163.com

**Keywords:** visual imaging, pixel-level spatiotemporal modulation, time-space ratio, feedback of CCD

## Abstract

At present, visual imaging is widely applied for surface defects such as bumps and scratches in the manufacture of precise parts with a highly reflective surface. However, the high light reflection and halo disturbance as a result of the illumination in visual imaging exert a direct influence on the accuracy of defect detection. In this regard, the present paper develops an adaptive illumination method based on space–time modulation for a visual imaging system. Furthermore, a digital micro-mirror device (DMD) is employed to realize the pixel-level spatiotemporal modulation of illumination. Then, in combination with the illumination intensity feedback of charge coupled device (CCD), the time-space ratio is adjusted automatically to achieve adaptive uniform illumination and effectively suppress the high light reflection and halo disturbance of highly reflective surfaces. The experimental results show that, in terms of restraining high light disturbance, the visibility and accuracy of visual imaging are improved.

## 1. Introduction

A highly reflective surface refers to the surface of parts which are characterized by extremely strong reflectivity—almost mirror reflection—as well as the clear reflection of object images. Rotary parts, such as bearing steel balls, rollers and precision shafts, are widely used in military and civil fields, where the accurate detection of surface defects plays a vital role. At present, visual fast measurement is frequently employed for detection. However, in the photographing process of high-reflective surface images, an urgent problem arises: the specular reflection disturbance, caused by a highly reflective surface, leads to the brightness distortion of images and the covering or destruction of defect information. Along the normal direction of the CCD target in particular, a wide range of light concentrations appears, which seriously affects the effectiveness and validity of defect detection.

In an attempt to reduce the halo in the normal direction of CCD targets, scholars at home and abroad have conducted a large amount of research. Wang Yiwen, et al. weakened the reflection of a steel ball surface by putting the measured object into 10 ^#^ aviation hydraulic oil medium [1]. With the same purpose, Zhao Yeling, et al. employed lighting boxes [2], and Do Yongtae, et al. installed a plastic shade with a transmittance of 70% around the ball [3]. All these schemes contribute to the reduction of haloes. However, saturated spots still exist as the brightness of the whole image decreases, and the black area, owing to the projection of CCD lens in the captured image, makes it difficult to distinguish the image. There are also some scholars who have worked from the perspective of image processing to eliminate reflection, such as multi image fusion technology [4], but this will increase the complexity of subsequent image processing algorithms, which is not suitable for rapid detection. With regard to the mirror reflection characteristics of smooth spherical surfaces, Ng et al. [5] and Le Jing, et al. [6] adopted the method of photographing the reflective band image modulated by the surface topography to realize defect detection, but the detection accuracy is affected by the scanning interval, and only achieves a low speed. Our strategy of the visual imaging of a smooth surface to eliminate high reflection disturbance is motivated by the consideration of improving the reliability and accuracy and guaranteeing the efficiency of defect detection.

Given the above experimental results [7,8,9], great importance, at present, is attached to the design of a reasonable lighting method for this kind of metal surface with strong reflectivity on the premise of good defect visibility. Based on the uniform light source, the present study develops a visual imaging platform for high reflection rotating surface defects. With the uniformity of light sources, the DMD control technique based on the field-programmable gate array (FPGA) is used to achieve micro-mirror modulation. Combined with the designed illumination stripe algorithm, the high-brightness reflection and halo disturbance of the high-reflective surface are effectively suppressed with the enhancement of defect visibility and an improved accuracy of defect visual imaging.

## 2. The System for Visual Imaging

### 2.1. Visual Imaging Principle

**Figure 1:**
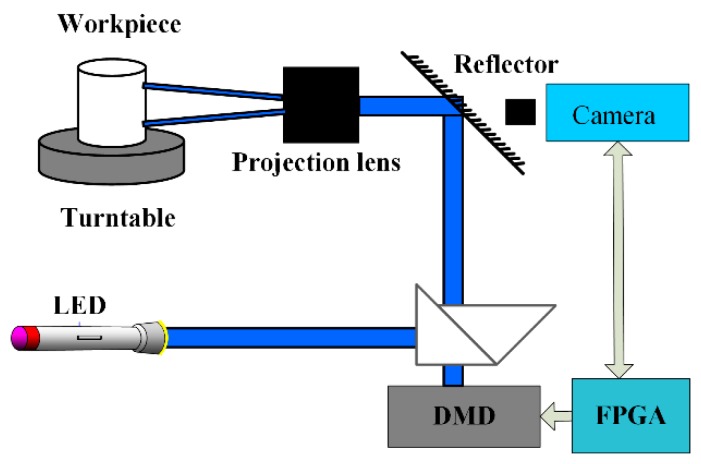
Defect detection system DMD: digital micro-mirror device; FPGA: field-programmable gate array.

The overall design of the visual imaging of an adaptive illumination method is shown in Figure 1 First of all, a collimated LED light source is incident on the total internal reflection (TIR) prism, and the incident light is reflected by the triangular prism and illuminated vertically on the DMD. Then, the reflected light (adaptive stripes with spatiotemporal modulation) modulated by DMD is projected through a triangular prism, mirror and projection lens onto a workpiece with a high reflective surface, and a uniform illumination is formed on the workpiece surface to highlight the defect information. Finally, the image acquisition module is used to capture the defect information of the workpiece surface to be measured.

### 2.2. Composition of Adaptive Illumination System

#### 2.2.1. LED Light Source

The light sources mainly used in the illumination system are a common light source, laser light source and LED. The common light source consists of a super metal halide lamp, high-pressure mercury lamp, xenon lamp and halogen lamp [10,11,12]. Compared with the traditional light source, the LED light source is characterized by a long life, stable output, low price, etc. Moreover, the lifetime of the LED is more than 100,000 h, and the lifetime of the optical system is improved accordingly. Therefore, the high-power LED is used in the illumination path to ensure the brightness of the figure projected on the surface of the workpiece under the light environment.

#### 2.2.2. Spatiotemporal Light Modulation

**Figure 2:**
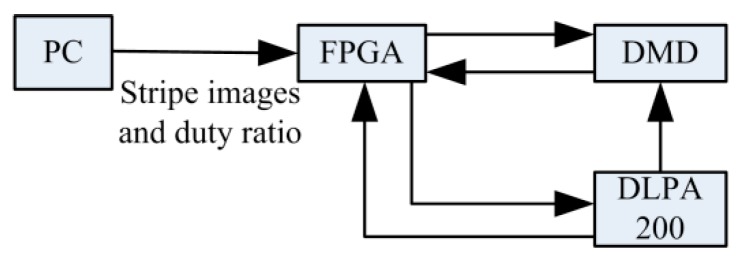
System block diagram of the FPGA control DMD. DLPA: digital light processing A.

With regard to the scheme design of the illumination path, as shown in Figure 2, the adaptive illumination with space-time modulation is realized by the pixel-level dimming technology of DMD. Spatial light modulation (SLM) technology can achieve the intensity modulation of incident light according to the computer control of the on/off state of each micro-mirror and generate various high-contrast accurate figures. The projection image, generated by DMD [13,14,15,16,17,18], is composed of “on” and “off” for the micro-lens. Generally speaking, the micro-mirror array on the DMD chip is divided into X direction and Y direction, which corresponds to two-dimensional detection points on the surface of the workpiece with high reflection rotation.

The DMD of the core digital micro-mirror of the spatial light modulation part is 0.7 inches, with a resolution of 1024 × 768 and a size of each micro-mirror of 13 × 13 μm. Each micro-mirror represents a pixel, with the distance between the micro-mirrors being less than 1 μm, and has a positive and negative deflection angle of 12°, which corresponds to the “on” state and “off” state, respectively. The control of each micro-mirror in DMD is realized through the combination with FPGA.

#### 2.2.3. Image Acquisition

**Figure 3:**
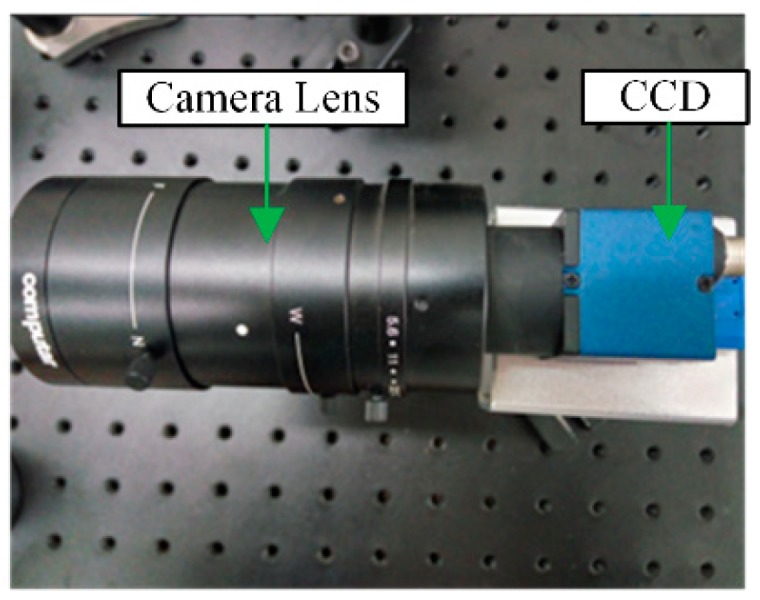
The composition of the image acquisition system.

Based on the comparison between CCD and complementary metal oxide semiconductor (CMOS)-hotosensitive chips, a digital camera equipped with a CMOS photosensitive chip, USB 3.0 and integrated general purpose input output (GPIO) interface is selected for data transmission, which leads to good robustness and a low deformation rate of the acquisition module, as shown in Figure 3.

## 3. A Visual Imaging Method of Adaptive Illumination

According to the characteristics of the rotating surface of a cylinder with high reflection of the measured workpiece, the illumination path projects straight stripes. In the case of the width of a dark stripe larger than the minimum detection defect, projected by the lighting system on the surface of the workpiece, the dark stripe will cover up the defect, leading to the missed detection of the defect. Therefore, the uniformity of stripes is required for the stripes projected on the surface of the workpiece.

### 3.1. Uniform Straight Stripe Algorithm

**Figure 4:**
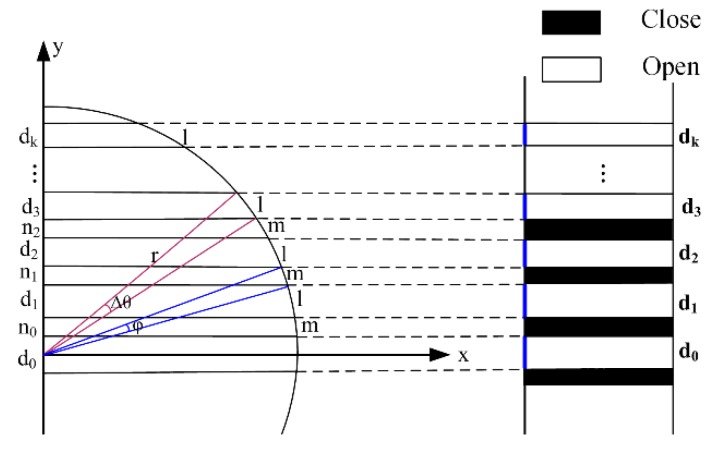
The schematic diagram of the stripe algorithm.

A two-dimensional rectangular coordinate system x-y is established with the center of the cylinder as the coordinate origin, as shown in Figure 4. According to the minimum defect detection and projection model, 1 is the arc length of bright stripes, m is the arc length of dark stripes and R is the radius of a cylinder. In the rectangular coordinate system, the first bright stripe arc length is divided equally by the X axis. In addition, *d*_0_~*d*_i_ refers to the width of bright stripes projected by the established model and *n*_0_~*n*_i_ refers to that of dark stripes. ∆*θ* is the arc angle corresponding to the arc length of bright stripes and φ is the arc angle corresponding to the arc length of dark stripes.

From the formula of arc length:

(1) Δθ=1/r

(2) φ=m/r

*d*_0_ refers to the width of the first bright stripe, which is:

(3) $$d_{0}=2r\;sin\left(\frac{\Delta \theta }{2}\right)=2r\;sin\left(\frac{l}{2r}\right)$$

where *θ*_0_ is the sum of a half of the arc angle corresponding to the first bright stripe and arc angle corresponding to the first dark stripe, which is:

(4) $$\theta_{0}=\frac{m}{r}+\frac{\Delta \theta }{2}=\frac{2m+l}{2r}$$

where *n*_0_ refers to the width of the first dark stripe projected by the established model on the examined workpiece, which is:

(5) $$n_{0}=r\;sin\theta_{0}-\frac{d_{0}}{2}=r\left[sin\left(\frac{2m+l}{2r}\right)-sin\left(\frac{l}{2r}\right)\right]$$

By analogy, we can obtain the width *d_i_* of the *i*th bright stripe projected by the established model on the workpiece:

(6) $$\begin{array}{c} d_{0}=r\left[sin(\theta_{0}+\Delta \theta +\left(i-1\right)\left(\Delta \theta +\varphi \right)\right)-sin\left(\theta_{0}+\left(i-1\right)\left(\Delta \theta +\varphi \right)\right)]\\ =r\left[sin\left(\frac{2m+3l}{2r}+\left(i-1\right)\left(\frac{m+l}{r}\right)\right)-sin\left(\frac{2m+l}{2r}+\left(i-1\right)\left(\frac{m+l}{r}\right)\right)\right] \end{array}$$

where *i* = 1, 2, 3,…, k.

In the same way, the width *n_i_* of the *i*th dark stripe projected by the established model on the workpiece can also be calculated as follows:

(7) $$\begin{array}{c} n_{i}=r\left[sin(\theta_{0}+\Delta \theta +\left(i-1\right)\left(\Delta \theta +\varphi \right)\right)-sin\left(\theta_{0}+\left(i-1\right)\left(\Delta \theta +\varphi \right)\right)]\\ =r\left[sin\left(\frac{2m+l}{2r}+i\left(\frac{m+l}{r}\right)\right)-sin\left(\frac{2m+3l}{2r}+\left(i-1\right)\left(\frac{m+l}{r}\right)\right)\right] \end{array}$$

where *i* = 1, 2, 3, …, k.

### 3.2. Simulation of Uniform Stripe Algorithm

It is by no means easy to use optical design software to simulate the working principle and projection of a DMD micro-mirror [19]. In the present paper, DMD, with a resolution of 1024 × 768, is adopted to simulate 786, 432 mirrors, which poses a difficult problem for the optical design software. On this basis, in an attempt to verify the rationality of the stripe selection and the correctness of the uniformity algorithm, this study—drawing on Tracepro software—proposes DMD for the simulation of the stripe light source.

**Figure 5:**
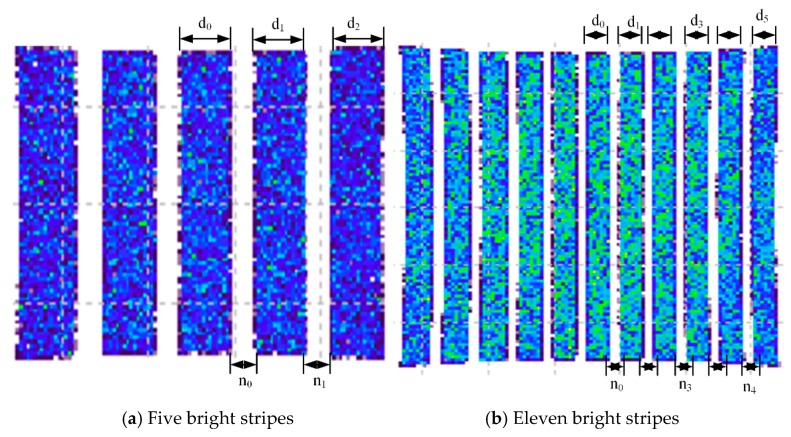
Simulation of the stripe illumination map of a cylindrical surface.

For the verification of the uniformity algorithm of stripes, in the first group, it is assumed that the radius of the cylinder is 40 mm, and five bright stripes with equal arc lengths are projected on the surface of the cylinder with a central angle of 2° but of 1° of dark stripes. Based on the focal length and magnification of the convex lens, the width of the DMD stripe light source is controlled and the illumination diagram of the cylindrical surface is as shown in Figure 5a, where the theoretical value of d and the actual width of each stripe are presented in Table 1. The second group assumes that 11 bright stripes with equal arc lengths are projected on the surface of the cylinder. The central angle of bright stripes is 1°, while the angle of dark stripes is 0.5°. According to the focal length and location of the convex lens, the width of the DMD stripe light source is controlled and the illumination diagram of the cylindrical surface is as shown in Figure 5b, where the theoretical value of d and the actual width of each stripe are as presented in Table 2.

As Table 1 and Table 2 indicate, the data obtained by the uniform stripe algorithm are almost consistent with those by simulation, and the maximum deviation is less than 17 µm of the distance between the micro-mirrors of DMD, which verifies the uniform stripe algorithm.

### 3.3. Visual Imaging of Adaptive Illumination of Light Intensity

According to the scheme of the illumination detection system, the stripe defect pattern on the surface of the workpiece is captured by a digital camera. When the parallel stripes illuminate the surface of a revolving object, there is an inconsistency in the scattering characteristics as a result of the reflection angle and surface quality at different positions, and the difference is also shown in the light intensity obtained by the digital camera, which easily produces high-brightness reflection and halo disturbance, leading to the poor manifestation of defects. Therefore, it is necessary to adjust the light intensity projected on the surface of the inspected workpiece according to the scattering model. The bi-directional scattering distribution function (BSDF) can describe the optical scattering characteristics of the target surface vividly and accurately, which is further defined as the scattering intensity generated by the incident light intensity, specifically expressed as follows:

(8) $$BSDF\left(\theta_{i},\phi_{i},\theta_{s},\phi_{s}\right)=\frac{dL_{s}\left(\theta_{s},\phi_{s}\right)}{dE_{i}\left(\theta_{i},\phi_{i}\right)}$$

**Figure 6:**
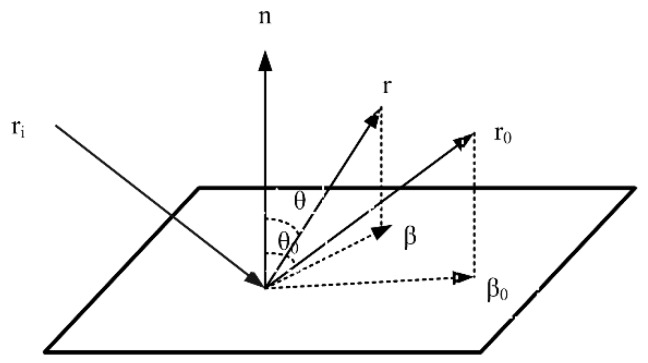
Schematic diagram of the bi-directional scattering distribution function (BSDF) model.

Figure 6 shows a schematic diagram of the BSDF model. *β*_0_ is the projection of unit vector *r*_0_, and *β* is the projection of unit vector r along the scattering direction. The modulus between the two |*β* − *β*_0_| warrants further investigation of BSDF.

The Harvey–Shack method presents a model suitable for most optical surfaces, which is mainly applied in the scattering caused by surface roughness. The model has the following characteristics. Owing to the isotropic scattering property, the surface roughness is smaller than the wavelength of light. The surface of the high-reflection rotary workpiece studied in this paper satisfies the scattering model. When BSDF is measured or evaluated in the incident plane—that is, when the scattering direction r is coplanar with the incident direction and the mirror direction—|*β* − *β*_0_| degenerates to |sin*θ* − sin*θ*_0_|, where *θ* is the angle between the normal and the scattering direction and *θ*_0_ is the angle between the mirror direction and the surface. Given the vertical incident of light on the surface, *θ*_0_ = 0, so |*β* − *β*_0_| = sin*θ*. The BSDF function model in the Tracepro software is the inverse power law model of ABg, and the specific expression is presented as follows:

(9) $$BSDF=\frac{A}{B+{\left|\vec{\beta }-{\vec{\beta }}_{0}\right|}^{g}}$$

**Figure 7:**
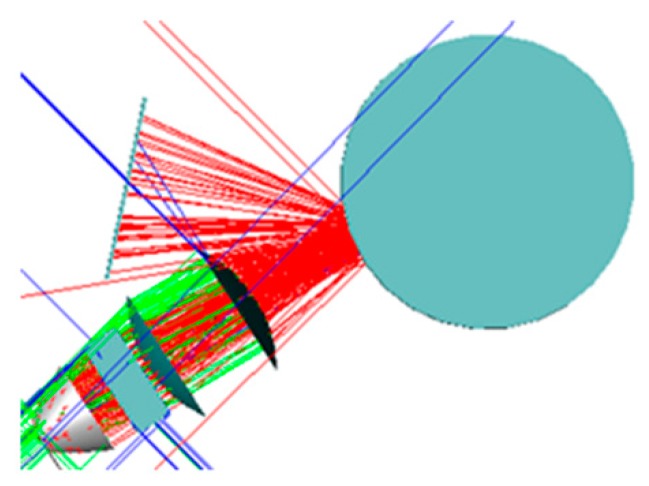
Ray tracing graph of scattering model.

The simulation of the scattering model is conducted in Tracepro software. Based on the uniform illumination of the LED light source, a cylindrical model with the radius of 40 mm is established, with the surface property of the AB_g_ scattering model. Supposing that the light number is 100,000, the wavelength is 455 nm, and the light source power is 3 w. The light tracing diagram is shown in Figure 7. In an attempt to realize the workpiece detection with different curvature radii in this paper, it can be found from the diagram of the scattering model that it is rather demanding and complicated to quantitatively calculate the scattering amount in all directions for each inspected workpiece. Besides, as a result of the characteristics of the defect location of workpieces, it is difficult to obtain the actual intensity property. Therefore, the present study, from the images captured by the digital camera, adopts an adaptive control technology based on the feedback mode to achieve the spatiotemporal modulation of stripe intensity.

First of all, according to the uniform fringe algorithm and the projection model, DMD is controlled by FPGA to illuminate the workpiece surface with a space-time duty ratio of *DR*_0_ = 50%. In addition, the intensity value of each stripe can be obtained by photographing the surface image of the workpiece with a digital camera. Moreover, the spatiotemporal duty ratio DRi of each stripe is acquired according to the stripe spatiotemporal adjuster, and the intensity of the stripe is modulated by DMD controlled by FPGA. The digital camera continues to collect images. The modulation comes to an end in the case of the relatively uniform intensity of stripes in the collected image or the visibility of defects. Considering the nonuniform intensity of stripes, the modulation is terminated when the uniform intensity or defect appears; otherwise, the time duty cycle for each stripe has to be reworked. The specific process is as follows:

(1) Firstly, according to the uniform straight stripe algorithm, the illumination stripes *P*_1_–*P*_N_, as well as the distribution of the workpiece, are calculated, where N is the total number of stripes.
(2) Suppose that the space-time duty ratio of each stripe is *DR*_i_ = 50 %; then, *i* = 1–N.
(3) Based on the space-time duty ratio of each stripe, the workpiece is illuminated by the time–space modulation stripe by the control of FPAG over DMD.
(4) The image of the workpiece in the illumination area is captured by a digital camera. The grey value RGBi, standard deviation σ of each stripe, the deviation *Err*_i_ = *RGB*_i_ − *Avg* (average grey value) and the maximum (Max) and minimum (Min) grey value of each stripe are calculated.
(5) The requirement of illumination uniformity can be determined according to the value of σ. If σ > [σ], the space-time duty ratio *DR*_i+1_ = *DR*_I_ + *Err*_i_/(Max − Min)∗K of each stripe should be revised, and the process should return to step (3); if σ <[σ], the adaptive modulation of light comes to an end.

K and [σ] are determined according to specific engineering conditions and empirical values, respectively.

## 4. Experiment Results

### 4.1. Experimental Platform

The above algorithm is validated experimentally in the following experimental environments: according to the detection illumination system introduced in Section 2 of this paper, the optical path devices are selected, as shown in Table 3.

### 4.2. Experimental Analysis

**Figure 8:**
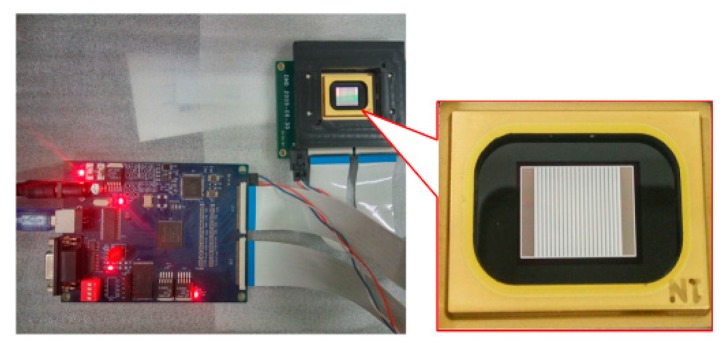
DMD stripe control results.

The cylindrical workpiece with a radius of 40 mm, as the experiment target, is placed in the optical path, undergoing two groups of tests. Based on the minimum defect size on the surface of a rotating cylinder with a high reflection, the arc lengths of bright and dark stripes are determined, the spacing of bright and dark stripes on DMD is determined, and DMD is modulated according to the calculated spacing width of bright and dark stripes. Figure 8 shows the stripe pattern of DMD in the experiment.

**Figure 9:**
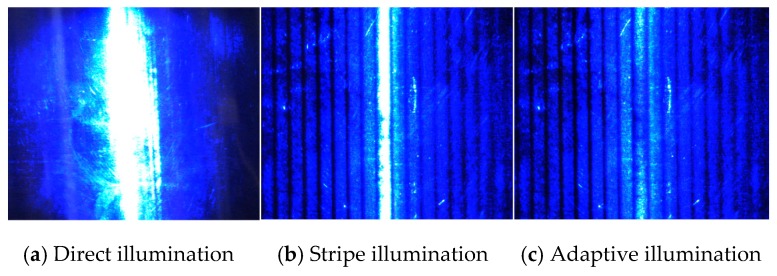
Different methods of illumination image capture results.

The measured cylindrical workpiece with a radius of 40 mm is properly placed in the experimental platform. First and foremost, the LED light source illuminates the cylinder workpiece being inspected, and the surface image of the cylinder with a high reflection is captured by the digital camera. As Figure 9a shows, the image contains large areas of halo and the over exposure of light intensity, and the defects are concealed. Then, the uniform straight stripe light is projected onto the surface of the cylinder under examination, and the surface image captured by the digital camera is shown in Figure 9b. Compared with the workpiece surface not illuminated by the stripe light, the halo phenomenon is obviously reduced, but the normal direction of the target surface of the digital camera is still overexposed, which causes the defects in the central bright stripe to be concealed. At last, illuminated by the adaptive modulation scheme of light intensity shown in Section 3 of this paper, the image captured by the digital camera is shown in Figure 9c, which eliminates the halo and light intensity overexposure phenomenon and causes the defects to appear completely.

In an attempt to further evaluate the effect of the optical system and defect visibility, the gray value of each stripe interval in the defective image captured by the digital camera is calculated. Figure 9b shows the defect image of the uniform stripe captured by the digital camera. In total, there are 16 stripes, and the data are shown in Table 4.

As Table 4 shows, the gray values of the 9th and 10th stripes on the target surface of the digital camera are much larger than those of the stripes on both sides, which correspond to the overexposure position of the light intensity phenomenon in the image of Figure 9b. This indicates that the gray value of the stripes can be used to evaluate the quality of the image and the visibility of the defects. Table 5 shows that the gray values of the 9th and 10th stripes on the target surface of the digital camera decrease obviously, which correspond to the location of the defect improvement in the image of Figure 9c It is proved that the adaptive illumination method based on the spatio-temporal modulation in this paper can effectively suppress the bright reflection and halo disturbance of a highly reflective surface and further improve the accuracy of defect detection.

In order to better evaluate the discreteness of the gray values of stripes, the standard deviation of the two groups of data is calculated. Table 4 presents the calculation of the gray value of each stripe and the standard deviation of data in the original defect graph, and we can obtain σ_1_ = 2.0 × 10^6^. We calculate the gray value of each stripe and the standard deviation of data in the modulated defect graph in Table 5, and we obtain σ_2_ = 0.8 × 10^6^. The standard deviation of the modulated data is approximately half of that before modulation, and the adaptive illumination method based on the spatio-temporal modulation in this paper is further explained. The effective suppression of bright reflection and halo disturbance on highly reflective surfaces improves the accuracy of defect detection, which also indicates that the smaller the standard deviation, the more uniform the light intensity of the collected image and the more accurate the defect detection.

## 5. Conclusions

In view of the surface defects such as bumps, scratches, and so on which may occur in the production process of precision parts with a highly reflective surface, the problems of light intensity overexposure and halo disturbance, which affect the accuracy of defect detection, arise in the employment of the main visual imaging methods. An adaptive optical illumination system based on DMD spatiotemporal modulation is designed which takes a digital micro-mirror device as a pixel level dimming core. Furthermore, the introduction of a collimated light source and uniform stripe algorithm can effectively suppress the intensity overexposure and halo disturbance in the process of high-reflection surface detection. The experimental results show that the system enhances the defect visibility on the premise of suppressing high light disturbance and meets the visual imaging requirements of different high-reflection surfaces. However, the system itself also manifests some shortcomings, such as the low brightness of the uniform stripe edge caused by the insufficient power of the LED light source in the experiment.

## Figures and Tables

**Table 1:** Theoretical and measured values of illumination stripe width.

Stripe No.	Theoretical Value/mm	Measured Value/mm	Err/mm
1	0.698	0.702	−0.004
2	0.349	0.321	0.028
3	0.697	0.684	0.013
4	0.348	0.336	0.012
5	0.694	0.704	−0.010

**Table 2:** Theoretical and measured values of illumination stripe width.

Stripe No.	Theoretical Value/mm	Measured Value/mm	Err/mm
1	0.349	0.357	−0.008
2	0.175	0.165	0.010
3	0.349	0.361	−0.012
4	0.174	0.182	−0.008
5	0.349	0.339	0.010
6	0.174	0.190	−0.016
7	0.348	0.357	−0.009
8	0.174	0.185	−0.011
9	0.347	0.350	−0.003
10	0.173	0.163	0.010
11	0.346	0.331	0.015

**Table 3:** The selection of optical devices. TIR: total internal reflection.

Device	Device Type
Illuminant	LED (455 nm)
Prism	TIR
DMD	0.7 Inch, 1024 × 768
Projection lens	F = 150 mm

**Table 4:** Gray value each stripe of uniform stripe illumination image.

Stripe No.	Gray Value (10^6^)	Stripe No	Gray Value (10^6^)
1	0.7	9	8.1
2	1.0	10	5.9
3	1.1	11	2.8
4	1.1	12	1.3
5	1.0	13	1.4
6	1.6	14	1.2
7	1.7	15	1.1
8	1.9	16	8.8

**Table 5:** Gray value for each stripe of the adaptive stripe illumination image.

Stripe No.	Gray Value (10^6^)	Stripe No	Gray Value (10^6^)
1	0.7	9	3.5
2	1.0	10	3.2
3	1.0	11	2.7
4	1.0	12	1.3
5	1.1	13	1.4
6	1.7	14	1.2
7	1.8	15	1.1
8	1.9	16	0.8
